# Effectiveness of VA-ECMO plus intra-aortic balloon pump for cardiac shock in patients with type A aortic dissection: a case series

**DOI:** 10.1186/s13019-023-02405-z

**Published:** 2023-10-24

**Authors:** Hui Wu, Pengfei Chen, Jinhua Wei, Fengbo Pei, Mingjian Chen, Diming Zhao, Liqing Wang, Jundong Pu, Zujun Chen

**Affiliations:** 1 Cardiovascular surgery department, Fuwai Hospital, Chinese Academy of medical sciences and Peking Union Medical College, National Center for Cardiovascular Diseases, Beijing, China; 2 Cardiovascular surgery department, Dali Bai Autonomous Prefecture People’s Hospital, Dali, China

**Keywords:** Extracorporeal membrane pulmonary circulation, Intra-aortic balloon pump, Type a aortic dissection, Coronary artery bypass graft

## Abstract

Limited reports exist on the utilization of venoarterial extracorporeal membrane oxygenation (VA-ECMO) following aortic dissection surgery, possibly due to concerns regarding complications. This case series aimed to evaluate the effectiveness and safety of using VA-ECMO in combination with intra-aortic balloon pump (IABP) for managing postoperative cardiogenic shock in patients with type A aortic dissection (AAD). The study included nine patients with an average age of 57.0 ± 9.5 years. The patients underwent various surgical procedures, including coronary artery bypass grafting (CABG) and aortic root reconstruction. The results showed that the combined use of VA-ECMO and IABP was feasible and effective in managing postoperative cardiogenic shock in AAD patients. However, the in-hospital mortality rate was high, with six out of nine patients succumbing to the condition. Among the patients who received VA-ECMO plus IABP in the operating room, four were successfully weaned from VA-ECMO, and three survived with a mean follow-up of 20 months. The study also highlighted the potential risks of renal complications associated with VA-ECMO and IABP. The findings suggest that the combined therapy of VA-ECMO and IABP may be beneficial for patients who have difficulty weaning from cardiopulmonary bypass (CPB) after AAD surgery.

## Introduction

The cardiogenic shock is a severe condition with high mortality after surgical treatment for type A aortic dissection (AAD) [1]. It is reported that 1–4% of patients after cardiovascular surgery needed postoperative venoarterial extracorporeal membrane oxygenation (VA-ECMO) as temporary mechanical circulatory support [2]. However, there are limited reports on the utilization of VA-ECMO assistance following aortic dissection surgery, possibly due to concerns regarding VA-ECMO-related complications [3]. One of the disadvantages of VA-ECMO in ischemic heart disease is its potential to increase left ventricular (LV) afterload and/or reduce coronary blood flow due to retrograde blood flow [4]. This disadvantage is thought to be compensated by the concomitant application of intra-aortic balloon pump (IABP) through a counterpulsation mechanism [5]. However, the use of IABP is generally contraindicated in AAD patients due to concerns about potential complications such as implantation into the false lumen, aortic rupture, or ischemic syndrome, and there are limited case reports documenting its clinical experience in this patient population [6]. Therefore, the objective of this study is to explore the effectiveness and safety of utilizing VA-ECMO in conjunction with IABP for managing postoperative cardiogenic shock in AAD patients.

## Methods

Approval was obtained (no.2023 − 2005) from the Ethics Committee of FuWai hospital. Patient informed consent was waived by the Ethics Committee of FuWai hospital. From January 1, 2018 to December 31, 2020, a total of 735 AAD patients received surgical treatment in our institution. Nine patients (1.2%) received the combined therapy of VA-ECMO and IABP for severe cardiac shock after the operation. The main indications for VA-ECMO included circulatory instability that occurs during or immediately after the discontinuation of the cardiopulmonary bypass after the initial operation and postoperative cardiac failure or cardiac arrest.

The clinical criteria of cardiogenic shock are defined as follows: hypotension, systolic blood pressure (SAP) < 80 mm Hg, mean arterial pressure (MAP) < 60 mm Hg; despite the best supportive measures, such as IABP, drugs, nitric oxide and phosphodiesterase inhibitors, still showing signs of renal failure (urine volume < 20 mL/h); anaerobic metabolism and metabolic acidosis (pH < 7.3, lactic acid level > 3.0 mmol/L). The hemodynamic standard is that the cardiac index (CI) is less than 30 mL/s/m2 and the pulmonary capillary wedge pressure is at least 20 mmHg.

### IABP implantation procedure

After puncturing the femoral artery and confirming that the needle tip was in the true cavity through blood pressure measurement or blood gas analysis, an ultra-smooth guide wire was implanted with the guidance of fluoroscopy in hybrid operating room. TEE was used to certify the position of guide wire. A 6 F sheath was then implanted through the ultra-smooth guide wire, and the location of sheath was also certified by TEE. IABP guide wire was implanted along with the sheath after removal of ultra-smooth guide wire. IABP was then implanted through the guide wire with confirmation of TEE again.

### VA-ECMO management

The VA-ECMO system is implanted with complete heparinization, and the activated clotting time (ACT) must be kept above 300 s. After establishing a complete VA-ECMO flow, unless there is continuous clotting or bleeding, ACT should be 140–180 s. In the first 24–48 h, VA-ECMO flow should be adjusted appropriately to maintain CI at 40 mL/s/m2, to keep mixed venous oxygen saturation (SvO2) at about 70% and MAP at 60–65 mmHg. Check the oxygenator twice a day to detect thrombosis as early as possible. After 48 h, daily hemodynamic, clinical and echocardiographic measurements were taken to evaluate the cardiopulmonary recovery to determine the best withdrawal time. When SvO2 ≥ 70%, hematocrit is 30–35%, there is no bleeding, cardiac tamponade or left heart dilation, left ventricular ejection fraction (LVEF) ≥ 35% and blood lactate level is normal, the withdrawal procedure should be started with caution. With continuous monitoring of hemodynamic and respiratory variables, the flow rate is gradually reduced to approximately 1 L/min. If there are signs of insufficient perfusion during VA-ECMO withdrawal, it should be increased to full flow again to extend the VA-ECMO support time. If the patient is hemodynamically stable under the minimum VA-ECMO flow and the myocardial function as assessed by echocardiography has recovered well, the VA-ECMO is withdrawn and the IABP is retained for further evaluation.

## Result

### Baseline characteristics of AAD patients treated with VA-ECMO plus IABP

The average age of patients was 57.0 ± 9.5 years old, of which 3 were female (33.3%). Table 1 summarized the basic clinical characteristics of this case series. Four patients had medical history of coronary heart disease and received the treatment of percutaneous coronary intervention (PCI). Two patients had iatrogenic AAD during the PCI procedure and suffered from preoperative cardiac arrest. One patient had the medical history of ascending aorta replacement due to type A aortic dissection.

**Table 1:** The baseline characteristics of AAD patients treated with VA-ECMO plus IABP

	IABP and ECMO (n=9)
Age (years)	57.0 ± 9.5
Female gender	3(33.3)
Comorbidity	
CHD	4(44.4)
Hypertension	5(55.6)
Hyperlipidemia	4(44.4)
BAV	2(22.2)
Marfan syndrome	0(0.0)
Medical history of cardiovascular surgery	1(11.1)
Medical history of PCI	4(44.4)
Current smoker	5(55.6)

Data are presented as the mean ± SD or n (%). AAD: type A aortic dissection; ECMO: extracorporeal membrane oxygenation; Intra-aortic balloon pump; CHD, coronary heart disease; BAV, Bicuspid aortic valve; PCI, percutaneous coronary intervention

### Clinical characteristics of AAD patients treated with VA-ECMO plus IABP

Acute AAD was presented in 7 patients, including 6 patients with DeBakey I AAD and one patient with intramural hematoma of the aorta (Table 2). The other 2 patients had chronic AAD with DeBakey II type. Among the 9 patients, 2 patients had preoperative comorbidity of coronary atherosclerotic stenosis, 2 patients had the involvement of coronary artery by dissection, and 3 patients presented with the co-exited condition of coronary atherosclerotic stenosis and coronary artery involvement by dissection. Table 2 also listed clinical presentation of these patients, including the echocardiographic results, the involvement of branch vessels by dissection, and preoperative creatinine and platelet level.

**Table 2:** Clinical presentation of AAD patients treated with VA-ECMO plus IABP

	IABP and ECMO(n=9)
Acute AAD	7(77.8)
DeBakey I	6(66.7)
IMH	1(11.1)
Iatrogenic AAD	2(22.2)
AAD presentation	
Chest pain	7(77.8)
Back pain	2(22.2)
Cardiac arrest	2(22.2)
Coronary artery involved by dissection	5(55.5)
CAS	5(55.5)
Diameter of aortic sinus	42.2 ± 8.9
DAA (mm)	43.8 ± 7.9
EF (%)	56.7 ± 7.4
LVEDD (mm)	51.7 ± 8.1
Creatinine (µmol/l)	86.9 ± 23.3
Platelet (×10^9^)	214.1 ± 78.2

Data are presented as the mean ± SD or n (%). AAD: type A aortic dissection; ECMO: extracorporeal membrane oxygenation; Intra-aortic balloon pump; IMH, intramural hemorrhage and hematoma; CAS, Coronary atherosclerotic stenosis; DAA, Diameter of ascending aorta; EF, ejection fraction; LVEDD, Left ventricular end diastolic diameter

### The surgical procedure of AAD patients with VA-ECMO plus IABP

Coronary artery bypass grafting (CABG) was performed in 7 patients, including 2 patients with scheduled CABG, 3 patients receiving CABG due to with difficulty weaning from the cardiopulmonary bypass (CPB), and 2 patients receiving CABG during the second thoracotomy (Table 3). Three patients had the CABG of single right coronary artery, and 4 patients had the CABG of both the right coronary artery and left anterior descending artery. During the operations, 4 patients (44.4%) received aortic root reconstruction (Bentall) simultaneously. Seven patients received total arch replacement with frozen elephant trunk (FET) procedure and 2 patients undertook partial arch replacement procedure. Table 3 listed the mean operation time of these patients, including cardiopulmonary bypass time, cardiac arrest time, deep hypothermia circulatory arrest time, and the lowest temperature.

**Table 3:** The treatment of AAD patients with VA-ECMO plus IABP

	IABP and ECMO (n=9)
Emergency surgery	7(77.8)
CABG	7(77.8)
Scheduled CABG	2(22.2)
CABG due to difficulty weaning from CPB	3(33.3)
CABG of single right coronary artery	3(33.3)
CABG of right coronary artery and left main	4(44.4)
Bentall	4(44.4)
Total arch replacement with FET	7(77.8)
Partial arch replacement	2(22.2)
CPB time (min)	382.6 ± 205.6
Cardiac arrest (min)	129.9 ± 49.2
HCA time(min)	8.2 ± 9.4
The lowest temperate(℃)	26.6 ± 2.2
The main reason for ECMO therapy	
Unable to wean from CPB	6 (66.7)
Postoperative cardiac failure	3 (33.3)
ECMO time(hours)	126.1 ± 96.7
ECMO pump flow max(L/min)	3.0 ± 0.5
ECMO cannulated vessels	
A side branch of the aortic graft	5 (55.5)
Femoral artery	4 (44.4)

Data are presented as the mean ± SD or n (%). AAD: type A aortic dissection; ECMO: extracorporeal membrane oxygenation; Intra-aortic balloon pump; CABG: coronary artery bypass graft; CPB: cardiopulmonary bypass; FET: frozen elephant trunk; HCA: hypothermic circulatory arrest

### Outcomes of AAD patients treated with VA-ECMO plus IABP

The modified IABP implantation were successfully performed in all 9 patients. There were no IABP-related complications. For the overall 9 patients, in-hospital death happened in 6 patients with the short-term mortality of 66.7% (Table 4). In 6 patients, VA-ECMO plus IABP were applied in initial operation due to difficulty weaning from the cardiopulmonary bypass (CPB). Among them, 4 patients were successfully weaned from VA-ECMO, and 3 patients survived until now with the mean follow-up of 20 months. One patient passed by because of the gastrointestinal bleeding 5 days after weaning from VA-ECMO. All 3 survivors presented with postoperative acute renal failure with the treatment of renal replacement therapy. Two of them discharged with normal renal function and one patient developed into chronic renal failure with routine dialysis. VA-ECMO and IABP were used as a salvage treatment in the other 3 patients due to postoperative cardiac failure or cardiac arrest happened in intensive care unit (ICU), but none of them survived after the removal of VA-ECMO. Table 4 listed the in-hospital outcomes of these patients, including average length of ICU stay and assisted ventilation.

**Table 4:** Outcomes of AAD patients treated with VA-ECMO plus IABP

	IABP and ECMO (n=9)
In-hospital death	6(66.7)
Tracheostomy	0(0.0)
Mid-term survivor	3(33.3)
Successful wean from ECMO	4(44.4)
Recovery from acute renal failure	2(22.2)
Mid-term dialysis	1(11.1)
Length of ICU (days)	11.6 ± 8.9
Length of ventilator use(hours)	206.4 ± 158.4

Data are presented as the mean ± SD or n (%). AAD: type A aortic dissection; ECMO: extracorporeal membrane oxygenation; Intra-aortic balloon pump; ICU: intensive care unit

## Discussion

Our study aimed to investigate the effectiveness and safety of VA-ECMO in conjunction with IABP for managing postoperative cardiogenic shock in AAD patients. The results showed that the combined use of VA-ECMO and IABP could be a feasible and effective strategy for managing postoperative cardiogenic shock in AAD patients, although the in-hospital mortality rate was high. The high mortality rate observed in our study is consistent with previous reports, indicating that postoperative cardiogenic shock in AAD patients is a severe condition with high mortality [2, 4, 5, 7–10]. However, it is noteworthy that among the six patients who received VA-ECMO plus IABP in the operating room due to difficulty weaning from the CPB, four were successfully weaned from VA-ECMO, and three survived until now with a mean follow-up of 20 months. This suggests that the combined use of VA-ECMO and IABP may be beneficial for patients who have difficulty weaning from CPB.

The application of VA-ECMO in the postoperative cardiogenic shock in AAD patients remains controversial, and the available evidence from the literatures is limited [11, 12]. However, some concluded that VA-ECMO provides a reasonable support for AAD patients affected by cardiac shock after surgery [5, 11]. Additionally, the IABPs are considered contraindicated in aortic dissection with the concern about misplacement within the false lumen and resultant extension of the dissection flap or aortic rupture. Only case reports have been published describing the use of IABPs to support cardiac function after surgery for type A aortic dissection [13, 14]. In our study, all patients successfully underwent IABP implantation without IABP-related complications. Actually, there is no contradiction regarding the contraindications of IABP. It is well-established that IABP is not used in patients with preoperative aortic dissection. However, there is currently no literature available reporting the progression of dissection due to the placement of IABP after aortic repair surgery. It suggests that with careful patient selection and meticulous surgical technique, the use of IABP in patients after AAD repair surgery may be safe and effective.

However, it is important to note that the use of VA-ECMO and IABP as a salvage treatment in patients with postoperative cardiac failure or cardiac arrest in the ICU was not successful in our study. One potential explanation for this could be that the timing of treatment was delayed, resulting in missed opportunities for earlier implementation of ECMO and IABP. It is possible that if these interventions had been initiated sooner, they could have provided significant benefits for these individuals. Further research and studies are needed to explore this possibility and determine the optimal timing for the use of ECMO and IABP in post AAD repair patients. In addition to the high mortality rate, another significant finding in our study was the incidence of postoperative acute renal failure in all three survivors, with one developing chronic renal failure requiring routine dialysis. This highlights the potential risk of renal complications associated with the use of VA-ECMO and IABP, which is consistent with previous reports [4]. This underscores the importance of careful patient selection and close postoperative monitoring for renal function in AAD patients receiving VA-ECMO and IABP.

The limitations of our study include the small sample size and the lack of a control group. Therefore, our findings should be interpreted with caution. Future studies with larger sample sizes and randomized controlled design are needed to validate our findings and to further investigate the effectiveness and safety of VA-ECMO in conjunction with IABP for managing postoperative cardiogenic shock in AAD patients.

## Conclusions

The combined support of VA-ECMO plus IABP could be considered as a salvage treatment for cardiogenic shock in AAD patients. The best benefit of the combined therapy may count for the patients with indications of difficulty weaning from CPB in the operating room.

## Data Availability

The data used to support the findings of this study are available from the corresponding author upon request.
